# Antipsychotic load during olanzapine mono- versus polytherapy and in relation to age

**DOI:** 10.1007/s00228-025-03947-y

**Published:** 2025-12-20

**Authors:** Vigdis Solhaug, Ragnhild Birkeland Waade, Lene Jansrud, Espen Molden, Elisabet Størset, Gudrun Høiseth, Marit Tveito

**Affiliations:** 1 Center for Psychopharmacology, Diakonhjemmet Hospital, PO Box 85, Vinderen, Oslo, 0319 Norway; 2 Department of Pharmacy, University of Oslo, Oslo, Norway; 3 Department of Forensic Sciences, Oslo University Hospital, Oslo, Norway; 4 Norwegian Center for Addiction Research, University of Oslo, Oslo, Norway

**Keywords:** Antipsychotics, Polypharmacy, Therapeutic drug monitoring, Pharmacokinetics, Aging

## Abstract

**Purpose:**

While antipsychotic monotherapy is generally recommended in treatment of psychotic disorders, polytherapy is frequently observed in clinical practice. We aimed to investigate the total antipsychotic load among patients treated with olanzapine in monotherapy vs. polytherapy, and how the antipsychotic load changed in relation to age.

**Methods:**

Patients with serum concentration measurements of olanzapine alone (monotherapy) or in combination with other antipsychotics (polytherapy) were retrospectively included from a therapeutic drug monitoring service. The antipsychotic load was measured as olanzapine dose and concentration equivalents. Patients were categorised into three age groups (18–49 years, 50–74 years and ≥ 75 years of age).

**Results:**

In total, 10 190 patients (54% males) were included, whereof 8 759 (86%) received olanzapine in monotherapy and 1 431 (14%) in polytherapy. The mean olanzapine dose equivalent was significantly higher in the polytherapy group compared to the monotherapy group (23.0 vs. 11.1 mg, *p* < 0.001). Likewise, the mean concentration equivalent was higher in the polytherapy group than in the monotherapy group (179 vs. 89.1 nmol/L, *p* < 0.001). When comparing patients ≥ 75 years with patients 18–49 years, the mean dose equivalents were 43% and 41% lower in the monotherapy and polytherapy group, respectively (both *p* < 0.001), whereas the respective concentration equivalents were only 11% (*p* < 0.001) and 10% (*p* = 0.141) lower.

**Conclusion:**

Patients receiving olanzapine in antipsychotic polytherapy had twice as high antipsychotic load compared to patients using olanzapine monotherapy, both in terms of olanzapine dose and concentration equivalents. Although the antipsychotic doses were reduced by age, the concentration levels remained similar.

## Introduction

Olanzapine is often the medication of choice in treatment of schizophrenia due to the effective reduction of overall positive and negative symptoms, according to large meta-analyses [1, 2]. However, the interpatient variability in clinical response of olanzapine treatment is large [3], including the risk of adverse effects [1, 2]. In the treatment of schizophrenia, antipsychotic monotherapy is usually recommended [4]. Although extensively debated [4–6], antipsychotic polytherapy is still common in clinical practice, where insufficient treatment response is the most common reason for prescribing multiple antipsychotics [4].

There is limited documentation from randomized controlled trials regarding the use of antipsychotic polytherapy [6, 7]. However, for olanzapine, one randomised controlled three-armed intervention study has been conducted, comparing olanzapine plus amisulpride with olanzapine plus placebo and amisulpride plus placebo in adult patients with schizophrenia [8]. After 8 weeks, the reduction in the Positive and Negative Syndrome Scale (PANSS) score (primary outcome) was higher in the group combining olanzapine and amisulpride than in the olanzapine plus placebo group, while the difference in PANSS score between the combination group and the amisulpride plus placebo group did not reach statistical significance. Side effects such as weight gain, increased waist circumference and sexual dysfunction were most frequent in the olanzapine plus amisulpride combination group, and hence the authors concluded that the increased clinical effect of amisulpride plus olanzapine has to be weighed against the increased risk of adverse effects [8]. An increased adverse effect burden of antipsychotic polytherapy is also described in a review by Gallego et al., including higher rates of parkinsonian side effects, hyperprolactinemia, sexual dysfunction, hypersalivation, sedation, cognitive impairment, and diabetes [9], which possibly reflect increased dose exposure, and hence increased risk of concentration-dependent adverse effects, compared to patients using antipsychotic monotherapy.

When comparing antipsychotic dosing, or calculating the total antipsychotic load, the use of chlorpromazine equivalents has been standard methodology quantifying overall dopamine antagonistic effect of first-generation (typical) antipsychotics [10]. However, since atypical antipsychotics exhibit less antidopaminergic potency with additional preference toward 5-HT_2A_ antagonism, newer methods have emerged to calculate equivalent doses of antipsychotics reflecting current clinical practice. Suggested methods include the classical mean dose method [11], the defined daily doses (DDD) method [12], the minimum effective dose method [13], a near maximum effective dose calculated from dose response studies [14] and consensus methods [15, 16]. The two most recent consensus studies comparing antipsychotic dosing employed the two stage Delphi method, in which psychiatrists from many countries were asked to estimate doses required to obtain an antipsychotic effect equivalent to 20 mg olanzapine during maintenance treatment of adult men with schizophrenia [15, 16]. Despite that a systematic review comparing methods for the estimation of antipsychotic dose equivalence concludes that no single method stands above the others [17], the consensus-based olanzapine dose equivalents [15, 16] appears to be most appropriate in current clinical context for measuring antipsychotic load in patients using antipsychotic polytherapy.

An important limitation with all dose equivalence calculations is the large intra- and interindividual variability in serum concentrations obtained at similar dosing. Several patient specific factors such as age, gender, comorbidity, smoking, pharmacogenetics and drug-drug interactions, as well as liver and kidney function, may contribute to this variability [18, 19]. Consequently, antipsychotic equivalent dose calculations will not reflect the actual antipsychotic load. To overcome this limitation, serum concentration data obtained during therapeutic drug monitoring (TDM) could be utilized to calculate antipsychotic equivalent concentrations and provide valuable insight into the true antipsychotic exposure.

To our knowledge, no studies have investigated the total antipsychotic load among patients using polytherapy compared to monotherapy, both in terms of doses and concentrations. By the use of TDM data, this observational study aimed to compare the total antipsychotic dose- and concentration equivalents in a patient population treated with olanzapine in monotherapy versus antipsychotic polytherapy, and in relation to age.

## Methods

### Subjects and samples

All TDM analyses of patients using oral olanzapine during the period January 2010 to June 2024 were included from the database at the Center for Psychopharmacology, Diakonhjemmet Hospital, (Oslo, Norway). For patients with multiple olanzapine TDM analyses, only the last measurement was included in the study to avoid patients being present in more than one group and to ensure steady-state condition. Furthermore, samples were excluded if *(1)* the olanzapine dose was below 5 mg, *(2)* the sampling time was outside the range of 10–30 h after the last dose intake or information on sampling time was lacking, *(3)* the serum concentration was above or below the quantification limits for the respective antipsychotics, and/or *(4)* patient’s age was below 18 years. Since a complete prescription profile was not available for the included patients, possible drug interactions with the antipsychotics were not assessed.

The polytherapy group consisted of patients who, in addition to olanzapine, had a simultaneous TDM measurement of one or more additional antipsychotics (Table 1). For the add-on antipsychotics, all doses and formulations were included, otherwise, the same exclusion criteria as listed above were applied for non-olanzapine antipsychotics. For antipsychotics administered as long acting injectables (LAI), samples with sampling time outside 10–35 or 20–35 days since last injection were excluded, for LAI administered every 2nd or 4th week respectively, and the daily dose was calculated as the injected dose divided by the number of days in the dosing interval of the LAI. Serum concentration measurements were assumed to be at steady state, as this is standard practice in the use of TDM.

**Table 1:** Number and frequencies of various antipsychotics used in combination with olanzapine

Antipsychotic medication	No. (%)
Quetiapine	458 (32.0)
Aripiprazole	260 (18.2)
Risperidone	197 (13.8)
Clozapine	146 (10.2)
Zuclopenthixol	120 (8.4)
Chlorprothixene	97 (6.8)
Levomepromazine	70 (4.9)
Amisulpride	41 (2.9)
Perphenazine	39 (2.7)
Haloperidol	37 (2.6)
Flupentixol	24 (1.7)
Ziprasidone	21 (1.5)
Lurasidone	16 (1.1)
Paliperidone	16 (1.1)

The study was approved by the Regional Committee for Medical and Health Research Ethics (2017/2191). This study is a part of the project “Ageing and antipsychotics”, registered in the Open Science Framework (OSF), registration DOI: 10.17605/OSF.IO/6BKEP.

### Serum concentration analysis

Serum concentration analyses of the antipsychotics were all performed by validated analytical methods for routine TDM analyses at the Center for Psychopharmacology, Diakonhjemmet Hospital, Oslo, Norway. For the two antipsychotics aripiprazole and risperidone, the total concentrations used included their active metabolites, dehydroaripiprazole and paliperidone, respectively. For all others, the parent compound was measured. During the time course of the retrospective data collection, the analytical assays had been modified due to renewal of the analytical instrumentation, but all the modifications were cross-validated. The accuracy of the analytical methods for the whole data collection period is ensured according to ISO 15,189 accreditation. Briefly, in the most recent Ultra-high-performance liquid chromatography high-resolution mass spectrometry (UHPLC-HRMS) method, the serum samples were prepared by protein precipitation using a Microlab Star pipetting robot (Hamilton, Reno, NV) in a semiautomated sample preparation procedure. The LC system was a Vanquish-UHPLC (Thermo Fisher Scientific, Waltham, MA), and chromatographic separation was performed by an XBridge BEH C18-column (2.6 μm, 2.1 × 75 mm; Waters, Milford, MA) using gradient elution at 35 °C with a 10 nM ammonium acetate buffer (pH = 4.8) for all the included antipsychotics, except zuclopenthixol. In a separate method,10 nM ammonium hydrogen carbonate buffer (pH = 8.1) was used for zuclopenthixol. Detection used a QExactive Orbitrap mass spectrometer (Thermo Fisher Scientific), operated in positive ionization mode, acquiring full scan data at a resolution of 70,000 within the 100- to 1500-Da scan range. All the calibration curves were linear (R^2^ > 0.99) and in validated ranges.

### Calculation of total olanzapine dose and concentration equivalents

A total olanzapine dose equivalent was calculated for each patient by use of olanzapine equivalents as estimated by the two international consensus studies of antipsychotic dosing performed by Gardner et al. [15] and McAdam et al. [16]. The olanzapine dose conversion factors (equivalency dose-ratio) for various antipsychotics are listed in Table 2. For patients using olanzapine monotherapy, the olanzapine dose was the olanzapine dose equivalent. For patients with antipsychotic polytherapy, each antipsychotic dose was converted by multiplying with the respective olanzapine dose conversion factor and then summed up to a total olanzapine dose equivalent for each patient.

**Table 2:** Doses of antipsychotics clinically equivalent to 20 mg olanzapine and their conversion factor (equivalency dose-ratio) according to Gardner et al. (1) and McAdam et al. (2). Median serum concentration of the different antipsychotics in the study population at the stated doses clinically equivalent to 20 mg olanzapine are given, and the calculated concentration conversion factor (equivalency concentration-ratio) for each antipsychotic

Antipsychotic medication	Clinically equivalent dose (mg)	Dose conversion factor	Median serum concentration (nmol/L) at clinically equivalent dose	Concentration conversion factor
Olanzapine	**20**	**1.00**	**128**	**1.00**
Amisulpride	600*	0.03	624	0.21
Aripiprazole	30	0.67	925	0.14
Chlorprothixene	500	0.04	44	2.91
Clozapine	400	0.05	1269.5	0.10
Flupentixol	10	2.00	14	9.14
Haloperidol	10	2.00	10	12.8
Levomepromazine	400	0.05	267	0.48
Lurasidone	111*	0.18	43	2.98
Paliperidone	5.36**	3.73	82	1.56
Perphenazine	24*	0.83	3	42.7
Quetiapine	700*	0.03	485	0.26
Risperidone	6	3.33	82	1.56
Ziprasidone	160	0.13	142.5	0.90
Zuclopenthixol	40*	0.50	51.5	2.49

*Doses differs from Gardner et al. and McAdam et al. Clinically equivalent doses of these antipsychotics are adjusted down to the nearest used dose in Norway. The dose conversion factor has been adjusted accordingly

**For paliperidone the nearest used dose in Norway is 150 mg long acting injection administered every 4^th^ week. The daily dose was calculated as the injected dose divided by the number of days in the dosing interval (150 mg/28 day = 5.36 mg/day)

The olanzapine concentration equivalents were calculated analogously to the dose equivalents. First, equivalency concentration-ratios for each antipsychotic were calculated by dividing the median serum concentration of olanzapine at 20 mg (128 nmol/L) by the median serum concentration of the respective antipsychotic at doses equivalent to 20 mg olanzapine. This equivalency concentration-ratio for each antipsychotic was used as the concentration conversion factor (Table 2) to convert the concentrations of the different antipsychotics to olanzapine concentration equivalents. For patients using olanzapine monotherapy, the olanzapine concentration was the olanzapine concentration equivalent. For patients with antipsychotic polytherapy, the concentration of each antipsychotic was converted by multiplying with the respective olanzapine concentration conversion factor and then summed up to a total olanzapine concentration equivalent for each patient.

### Analysed parameters and statistical analysis

The study outcome was the total olanzapine dose equivalents and concentration equivalents. These outcomes were assessed in relation to olanzapine monotherapy versus polytherapy (olanzapine + one or more other antipsychotics), and across three age groups (18–49 years, 50–74 years and ≥ 75 years of age).

The total olanzapine dose equivalents and concentration equivalents were ln-transformed before analysis to approximate normal distributions, which was assessed by visual inspection (qq-plot and histogram). Student t-test was used to compare the study outcomes between groups. All values were transformed to their original scale before presented. Mean group estimates are given with 95% confidence intervals (CI) and p-values. Statistical significance was considered as *p* < 0.05. STATA v.17.0 (StataCorp, TX, USA) software was used for the statistical analyses. The figure was developed using R v. 4.2.2 [20].

## Results

In total, data extracted from the TDM database amounted to 12 063 patients with olanzapine serum concentration measurements. 10 190 patients (54% males) met the criteria for inclusion. Among the included patients, 8 759 (86%) used olanzapine monotherapy and 1 431 (14.0%) used one or more antipsychotic medications in addition to olanzapine and constituted the polytherapy group (Table 3). In the polytherapy group, 1 327 patients used two antipsychotics, 97 patients used three and seven patients used four antipsychotics. Regarding the different age groups, the proportion of patients using antipsychotic polytherapy was highest among patients aged 18–49 years with 15.3% polytherapy, and lowest among the patients above the age of 75, with 7.1% polytherapy (Table 3).

**Table 3:** Population demographics

Variables	Monotherapy	Polytherapy
No. of patients (%)	8 759 (86.0)	1 431 (14.0)
Male/Female	4 709/4 050	782/649
Age, mean (min, max), year	49 (18,100)	46 (18,89)
Daily dose olanzapine, mean (min, max), mg	12.9 (5, 80)	13.6 (5, 75)
No. of patients in groups (%):		
18–49 y	4 562 (84.7)	823 (15.3)
50–74 y	3 157 (85.6)	529 (14.4)
≥ 75 y	1 040 (92.9)	79 (7.1)

In the study population, many different antipsychotics were used in combination with olanzapine. Quetiapine was the antipsychotic most often added to olanzapine therapy and used by 32% of the polytherapy group, followed by aripiprazole (18.2%) and risperidone (13.8%) (Table 1).

For the patients using olanzapine monotherapy the mean dose was 11.1 mg (95% CI 11.0, 11.2 mg). For the polytherapy group the mean olanzapine dose-equivalent was 23.0 mg (95% CI 22.4, 23.6 mg) (Table 4). The mean olanzapine serum concentration in the olanzapine monotherapy group was 89.1 nmol/L (95% CI 87.9, 90.3 nmol/L) and in the polytherapy group the mean olanzapine concentration-equivalent was 179 nmol/L (95% CI 174, 185 nmol/L) (Table 4). In Table 5, the mean olanzapine dose equivalents and concentration equivalents among patients using olanzapine monotherapy versus polytherapy, in the different age groups, are presented. Within each age group, both the dose equivalents and the concentration equivalents are about twice as high among patients using polytherapy compared to patients using olanzapine monotherapy (*p* < 0.001) (Table 5).

**Table 4:** Estimated mean total olanzapine dose equivalents and concentration equivalents among patients using olanzapine monotherapy versus polytherapy, and the Polytherapy/Monotherapy ratio (P/M ratio)

	Monotherapy	Polytherapy	*P*/M ratio	*p*-value
Olanzapine dose equivalents, mg (95% CI)	11.1 (11.0, 11.2)	23.0 (22.4, 23,6)	2.1	< 0.001
Olanzapine concentration equivalents, nmol/L (95% CI)	89.1 (87.9, 90.3)	179.0 (173.5, 184.6)	2.0	< 0.001

**Table 5:** Estimated mean total olanzapine dose equivalents and concentration equivalents among patients using olanzapine monotherapy versus polytherapy, in different age groups, and the Polytherapy/Monotherapy ratio (P/M ratio)

Group	Dose equivalents, mg (95% CI)				Concentration equivalents, nmol/L (95% CI)			
	Monotherapy	Polytherapy	*P*/M ratio	*p*-value	Monotherapy	Polytherapy	*P*/M ratio	*p*-value
18–49	12.7 (12.5, 12.9)	24.3 (23.6, 25.1)	1.9	< 0.001	92.6 (90.8, 94.5)	176.6 (169.5, 184.1)	1.9	< 0.001
50–74	10.6 (10.4, 10.8)	22.6 (21.7, 23.5)	2.1	< 0.001	86.5 (84.5, 88.5)	185.9 (176.7, 195.5)	2.1	< 0.001
≥ 75	7.2 (7.0, 7.4)	14.4 (12.8, 16.2)	2.0	< 0.001	82.2 (79.4, 85.2)	159.1 (140.4, 180.4	1.9	< 0.001

**Fig. 1:**
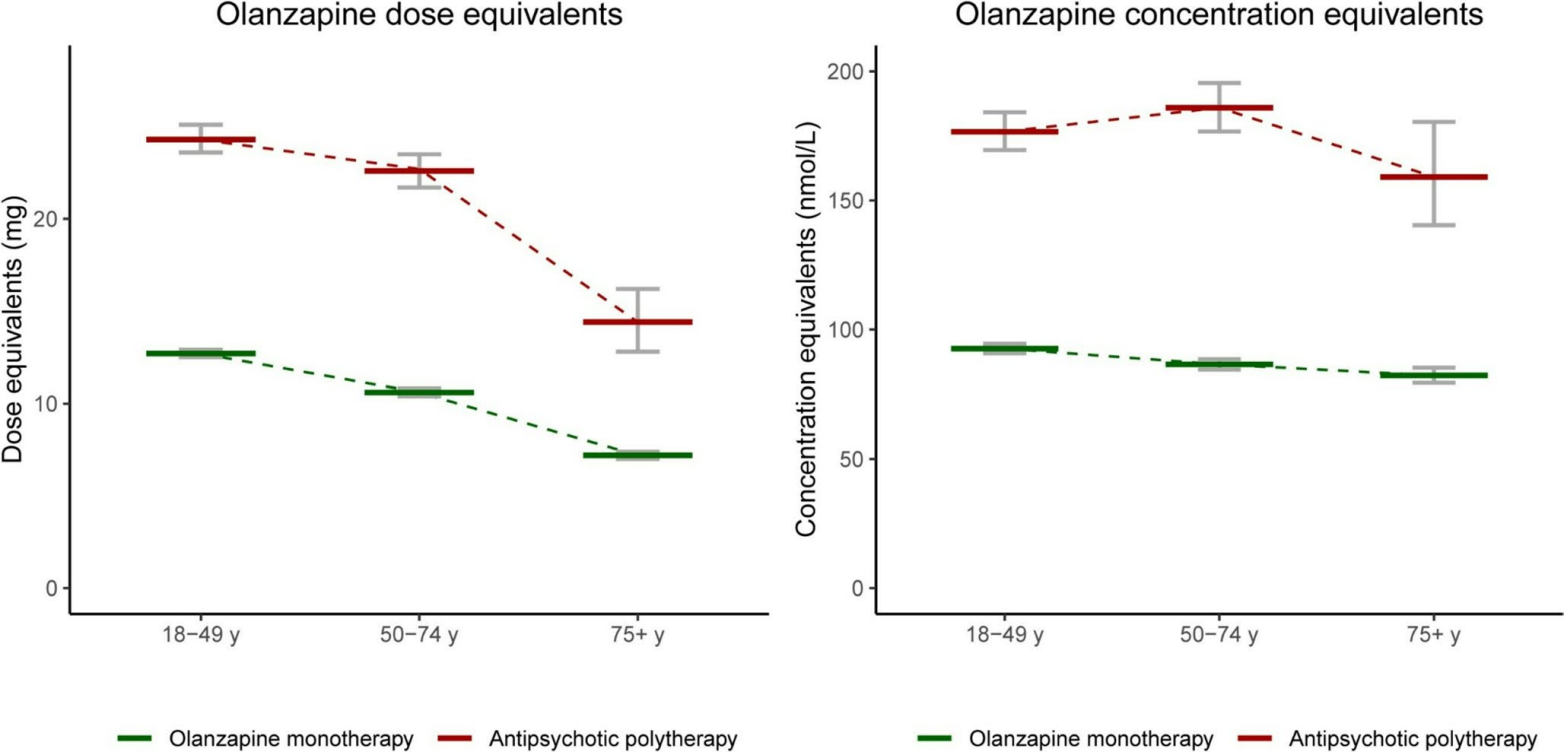
Olanzapine dose equivalents and concentration equivalents among patients using olanzapine monotherapy and antipsychotic polytherapy in different age groups. Points represent the estimated mean for each group. Error bars represent 95% confidence interval

The antipsychotic dose equivalents decreased more by age than the concentration equivalents. Figure 1 shows the stepwise reduction of the antipsychotic dose equivalents in the older age groups both among patients using monotherapy and polytherapy, whereas the antipsychotic concentration equivalents remain more similar across age groups. For patients using olanzapine monotherapy, the mean dose was 43% lower (*p* < 0.001) in the group of patients over the age of 75 compared to the 18–49 years group (Table 5). Also, among patients using polytherapy, the mean dose equivalent decreased by age and was 41% lower (*p* < 0.001) in the patients over 75 years compared to the 18–49 years group (Table 5; Fig. 1). In contrast, the concentration equivalents only marginally decreased by age. There was only an 11% reduction (*p* < 0.001) in the concentration equivalent in the group of patients ≥ 75 years compared to 18–49 years among patients using olanzapine monotherapy, whereas in the polytherapy group there was no significant reduction with age (−10%, *p* = 0.141) (Table 5; Fig. 1).

## Discussion

In this study, the total antipsychotic load was found to be twice as high among patients using antipsychotic polytherapy compared to patients using olanzapine monotherapy, both in terms of dose and concentration equivalents. To combine TDM data and olanzapine equivalents to calculate the total load of antipsychotics is, to our knowledge, not previously published. Additionally, this study demonstrated that while dose equivalents are reduced by around 40% among patients 75 years and older, the concentration equivalents, representing the total antipsychotic exposure, are only marginally decreased in the patient group ≥ 75 compared to the 18–49 year group.

The present study shows that the dose and concentration equivalents are twice as high among patients using antipsychotic polytherapy compared to monotherapy. This indicates that a second (or additional) antipsychotic is added to the first antipsychotic without reducing the existing drug dose, which may reflect inadequate treatment response to the first antipsychotic and/or more severe psychiatric illness in the polytherapy group. In the randomised controlled trial by Schmidt-Kraepelin et al. [8], the total dose (using olanzapine dose equivalents based on the same expert consensus study [15]) was significantly higher in the olanzapine plus amisulpride group than in the monotherapy groups, corresponding to 28.2 mg olanzapine equivalents in the combined group vs. 16.5 mg in the amisulpride group and 14.1 mg in the olanzapine group. This may explain the higher efficacy and increased adverse effects rate in the combination group. In two large observational studies [21, 22], comparing hospitalisation and treatment discontinuation among patients using antipsychotic polypharmacy versus monotherapy, the total antipsychotic doses were not provided. The importance of considering the doses when comparing antipsychotic monotherapy versus polytherapy has been emphasized by others [4, 23].

Age-related pharmacokinetic changes are the primary reason why antipsychotic concentration equivalents do not decrease in older patient groups to the same extent as dose equivalents. Decreased hepatic and renal function affect the clearance of antipsychotics as age increases, resulting in higher dose adjusted serum concentrations of olanzapine and other antipsychotics in the older patients [24–27]. Although antipsychotics are widely used in this population, their effects and tolerability have primarily been studied in younger patients. In a large meta-analysis that included 402 randomised controlled trials of antipsychotics for treating schizophrenia, the mean age of participants was 38 years [2]. When prescribing antipsychotics to older patients, the increased susceptibility to adverse effects must be considered. This class of medication also pose an increased risk of falls [28, 29], likely related to the dose dependent adverse reactions such as sedation, orthostatic hypotension and anticholinergic effects. Our study results underscore the pharmacokinetic reasons for dose reduction in older patients. Additionally, pharmacodynamic changes associated with aging may further increase this patient group’s sensitivity to elevated antipsychotic concentration, making them more vulnerable to dose-dependent adverse effects [30, 31]. This emphasizes the need for cautious prescribing practice for antipsychotic medications in older adults.

The method used to summarize antipsychotic doses and concentrations is exploratory and warrants discussion. To generate a measure of the total amount prescribed across different antipsychotic regimens, doses must be converted into a generic unit. Several methods have been suggested for estimating equivalent doses across antipsychotics [17]. In the present study, we used the consensus-based method established through a two-step Delphi survey process [15, 16], which encompasses all antipsychotics utilized by the study population. The treatment scenario for the two step Delphi method; to estimate doses of antipsychotics clinical equivalent to 20 mg olanzapine in the maintenance treatment of schizophrenia [15, 16], was considered appropriate for our study population. This decision was based on the fact that TDM data reflects real-world conditions and that patients in our study population had typically been treated for extended periods, as only their most recent TDM measurements were utilized.

The olanzapine equivalents are based on similarities in clinical effect rather than on tolerability [15]. The pharmacodynamic profiles differ among antipsychotics, especially among the atypical antipsychotics most commonly used today, leading to varying adverse effect profiles [2]. Consequently, when adding doses of antipsychotics converted to olanzapine equivalents, the risk profile will depend on which antipsychotics that are combined.

Although the summation of antipsychotic concentrations introduces some uncertainty, the total olanzapine concentration equivalents more accurately reflect the total antipsychotic exposure than the dose equivalents. TDM measurements represent the through concentrations at steady state [18]. By converting the concentrations of each antipsychotic to olanzapine concentration equivalents, we are able to summarise and give a rough estimate of the total antipsychotic exposure. Each antipsychotic has a drug-specific pharmacokinetic profile, but oral antipsychotics also share characteristics like good absorption form the gastrointestinal tract with maximum concentration within 1–6 h, high apparent volume of distribution, hepatic metabolism by CYP and UDP-glucuronyltransferase enzymes (except amisulpride), and linear pharmacokinetics at therapeutic doses [18]. Even if the olanzapine concentration equivalents may not provide a precise estimate of the sum concentration, this could be useful for investigating how patient factors affect pharmacokinetics of the total antipsychotic use, e.g. as in this study, how age affects antipsychotic exposure.

Other limitations of the study stem from its naturalistic setting. For the study population, there is no information about clinical outcome, comorbidity, weight, organ function, adherence, pharmacogenetics and concurrently used drugs (in addition to antipsychotics), as well as the indication for antipsychotic treatment. The study population consists of patients undergoing TDM monitoring, and even if TDM is widely used to monitor antipsychotic treatment in Norway [32], we cannot rule out the possibility of selection bias in the data. It is reasonable to assume that patients in the polytherapy group had more severe psychiatric illness and that comorbidity and comedication are more prevalent among the older patients and are factors that may have influenced the outcome measure. On the other hand, the naturalistic setting of the study represents the real-world use of antipsychotics. As the study population consists of nearly 9 000 patients using olanzapine monotherapy and more than 1 400 patients on antipsychotic polytherapy, we believe this study adds valuable knowledge about the antipsychotic load during monotherapy versus polytherapy and across age groups.

## Conclusion

Patients receiving antipsychotic polytherapy had twice as high antipsychotic load compared to patients using olanzapine monotherapy, both in terms of olanzapine dose and concentration equivalents. Although the antipsychotic doses were reduced by age, the concentration levels remained similar. This highlights the necessity for adequate dose reduction of antipsychotics in older adults to lower antipsychotic exposure and minimize the risk of adverse effects.

## Data Availability

The data that supports the findings of this study is available upon reasonable request to the corresponding author.
